# FROM ONCOLOGIST TO SURGEON - GENETICS IN COLORECTAL METASTASIS FOR SURGEONS

**DOI:** 10.1590/0102-6720202400075e1869

**Published:** 2025-02-10

**Authors:** Marília Polo Mingueti e SILVA, Jorge SABBAGA, Henry Luiz NAJMAN, Carlos David Carvalho NASCIMENTO, Ricardo Lemos COTTA-PEREIRA, João Eduardo Leal NICOLUZZI, Maria Ignez BRAGHIROLI, International Hepato-Pancreato-Biliary Association

**Affiliations:** 1 D’Or Institute for Research and Education - São Paulo (SP), Brazil; 2 D’Or Institute for Research and Education - Rio de Janeiro (RJ), Brazil; 3 Universidade de São Paulo, Faculty of Medicine, Cancer Institute - São Paulo (SP), Brazil; 4 Hepato-Pancreato-Biliary Unit, Hospital Angelina Caron - Curitiba (PR), Brazil

**Keywords:** Colorectal Cancer, Genetics, Neoplasms Metastasis., Cancer Coloretal, Genética, Metástase Neoplásica.

## Abstract

Colorectal cancer (CRC) is a common disease, with incidence in Brazil of 45,630 new cases per 100,000 inhabitants between 2023-2025. Risk factors for CRC can be evaluated between environmental and hereditary and their mode of presentation are classified as sporadic, inherited and familial. Sporadic disease is characterized by the absence of a family history and accounts for approximately 70% of all colorectal cancers, being more common over 50 years of age, with dietary and environmental factors implicated in its pathogenesis. Sporadic disease is characterized by the absence of a family history and accounts for approximately 70% of all colorectal cancers, being more common over 50 years of age, with dietary and environmental factors implicated in its pathogenesis. The percentage of patients with a true hereditary genetic predisposition is less than 10%, and these are related to the presence or absence of colonic polyps as an important manifestation of the disease. Non-polyposis diseases are known as hereditary non-polypomatous colorectal cancer (HNPCC) or Lynch syndrome, and polyposis diseases are familial adenomatous polyposis (FAP), MUTYH-associated polyposis (MAP), and hamartomatous polyposis syndromes (e.g., Peutz-Jeghers, juvenile polyposis, phosphatase and tensin homologue - PTEN, Cowden syndrome). These diseases are linked to a high risk of developing cancer. With the development of treatments in metastatic disease and the use of targeted therapies and their biomarkers, it was possible to evaluate them within clinical studies both in the primary tumor and in the correspondence of metastases.

## RESUMO

O câncer colorretal (CCR) é uma doença comum, com incidência no Brasil de 45.630 novos casos por 100.000 habitantes entre 2023-2025. Os fatores de risco para CCR podem ser avaliados entre ambientais e hereditários e seu modo de apresentação são classificados como esporádicos, hereditários e familiares. A doença esporádica é caracterizada pela ausência de histórico familiar e representa aproximadamente 70% de todos os cânceres colorretais, sendo mais comum acima de 50 anos de idade, com fatores dietéticos e ambientais implicados em sua patogênese. A porcentagem de pacientes com uma verdadeira predisposição genética hereditária é menor que 10%, e estes estão relacionados à presença ou ausência de pólipos colônicos como uma manifestação importante da doença. Doenças não polipóticas são conhecidas como câncer colorretal hereditário não polipomatoso (HNPCC) ou síndrome de Lynch, e doenças polipóticas são polipose adenomatosa familiar (FAP), polipose associada a MUTYH (MAP) e síndromes de polipose hamartomatosa (Peutz-Jeghers, polipose juvenil, fosfatase e homólogo de tensina - PTEN, síndrome de Cowden). Essas doenças estão ligadas a um alto risco de desenvolver câncer. Com o desenvolvimento de tratamentos em doenças metastáticas e o uso de terapias direcionadas e seus biomarcadores, foi possível avaliá-los em estudos clínicos tanto no tumor primário quanto na correspondência de metástases.

## INTRODUCTION

Colorectal cancer (CRC) is a common disease, with approximately 151,030 new cases each year in the United States^18^ and has a forecast incidence in Brazil of 45,630 new cases per 100,000 inhabitants between 2023-2025^17^. Risk factors for CRC can be evaluated between environmental and hereditary and their mode of presentation are classified as sporadic, inherited and familial

Sporadic disease is characterized by the absence of a family history and accounts for approximately 70% of all colorectal cancers, being more common over 50 years of age, with dietary and environmental factors implicated in its pathogenesis. The percentage of patients with a true hereditary genetic predisposition is less than 10%, and these are related to the presence or absence of colonic polyps as an important manifestation of the disease. Non-polyposis diseases are known as hereditary non-polypomatous colorectal cancer (HNPCC) or Lynch syndrome, and polyposis diseases are familial adenomatous polyposis (FAP), MUTYH-associated polyposis (MAP), and hamartomatous polyposis syndromes (e.g., Peutz-Jeghers, juvenile polyposis, phosphatase and tensin homologue [PTEN] Cowden syndrome). These diseases are linked to a high risk of developing cancer^3^. The third and least understood pattern is known as “familial” CRC, and is related to up to 25% of cases. These are those patients who do not fit into any genetic syndrome, but have a first-degree relative affected by the disease. Having a single first-degree relative diagnosed with CRC increases your risk by up to 1.7 times compared to the general population.

The mechanism of transformation of a normal colonic epithelium into invasive cancer is possibly related to specific genetic alterations, which can be inherited or acquired. Germline mutations are those that occur during or before fertilization of the ovum, and are then likely to be transmitted from parent to child. However, in cases where the mutation occurs spontaneously in the sperm, egg or zygote, the affected individual’s parents do not express the cancer phenotype, but future progeny may inherit the mutation. The most common alteration is that which occurs spontaneously in a cell during the growth or development of a given tissue or organ, and is called somatic mutation^9,16^.

The best-known evolution to colorectal cancer is the adenoma-carcinoma sequence, where the adenomas (adenomatous polyps) become dysplastic. They form when the usual mechanisms that regulate epithelial renewal are disrupted. Cell proliferation occurs at the base of the crypts, and as cells are continually moved towards the luminal surface, they stop proliferating and terminally differentiate. This orderly process is interrupted as the adenomas increase in size, becoming dysplastic and eventually reaching some invasive potential^5^.

In the 90’s, Fearon et al. described that germline or somatic mutations are necessary for malignant transformation and also about the accumulation of genetic mutations that characterize the biological behavior of the tumor^9^. Sporadic cancers result from the accumulation of multiple somatic mutations, while germline mutations are the basis of genetic syndromes (eg, familial adenomatous polyposis and Lynch syndrome)^3^. In addition to point mutations, other genetic alterations are implicated in human tumorigenesis and include altered DNA methylation and gene rearrangements, amplifications, overexpression, and deletions.

Although this pathway is the most studied and most common, there is evidence of an alternative route through serrated polyps, a group that encompasses a morphologic spectrum that includes hyperplastic polyps, mixed hyperplastic polyps/adenomas, and serrated adenomas^11^.

Because it is a heterogeneous disease that comprises several tumor phenotypes, colorectal cancer is characterized by several specific molecular and morphological alterations, which target tumor suppressor genes, oncogenes and those related to DNA repair mechanisms. As described above, depending on the origin of these mutations, CRC is classified as sporadic (70-75%), hereditary (5%) and familial (20-25%)^15^.

### Origin and progression of colorectal cancer

There are three main pathways that are involved in the origin and progression of CRC, described below:

1. Chromosomal instability (CIN);
2. Microsatellite instability (MSI);
3. CpG island methylation phenotype (CIMP), below we will describe its pathological, genetic and clinical characteristics^15^.

The most common genetic mechanism is chromosomal instability, accounting for 85% of all CCRs, characterized by the acquisition of consistent karyotypic variability, aneuploidy, chromosomal and subchromosomal aberrations, gene amplifications and loss of heterozygosity. The main one being the loss of heterozygosity in the loci of tumor suppressor genes. Another important feature of this subgroup is the association with the accumulation of mutations at the level of several oncogenes, including KRAS (*Ki-ras2 Kirsten rat sarcoma viral oncogene homolog*) and BRAF, and tumor suppressor genes such as APC (adenomatous polyposis coli) and TP53. Due to these characteristics, a meta-analysis demonstrated that this profile is associated with a worse prognosis^20^.

The second most common mechanism is the CIMP pathway - colorectal tumors that have a particularly high frequency of methylation of some CpG islands (in which a cytosine [C] base is immediately followed by a guanine [G] base that are linked by a phosphodiester bond [CpG]), responsible for 20-30% of all RCCs and is more frequent in the proximal colon (30-40%) and more rarely found in the distal colon (3-12%). This defect may result in hypermethylation of the promoter region of DNA repair enzymes such as MLH1 and silencing of gene expression. Activating mutations in the BRAF gene, mostly in the V600E codon, occur almost exclusively in MSI-H, CIMP+ tumors that do not carry any mutations in the KRAS gene^21^.

Finally, the mechanisms for microsatellite instability involve several recurrent changes in the microsatellite zone, without apparent structural and numerical changes in the genome. Approximately 15% of all RCCs have a high frequency of MSI due to germline mutations in the mismatch repair system (MMR) or somatic inactivation by hypermethylation of the MLH1 gene promoter^4^.

### Colorectal cancer molecular subtypes

It was through an analysis of gene expression, obtained in thousands of cases of CRC, that a classification for colon cancer was proposed, based on four main molecular consensus subtypes (CMS), CMS1 to CMS4. Below we will describe the main characteristics of each subtype^10^.

The CMS1 group (MSI immune subtype, 14%) is genetically characterized by hypermutation, hypermethylation, BRAFV600E mutations (40% of tumors) and mainly by significant infiltration of the tumor microenvironment by immune cells, particularly T lymphocytes (cytotoxic CD8+ and CD4+ T helper) and natural killer lymphocytes. The most frequent mutations in these tumors are in the APC gene (35%), TP53 (30%) and KRAS (25%), other possible mutations are in the MSH6, RNF43, ATM, TGFBR2, BRAF and PTEN genes. Its origin is more commonly described through precursor lesions with serrated histology, in proximal regions of the colon and has an intermediate prognosis, being poor after relapse^10^.

The canonical subtype, better known as CMS2, corresponds to 37% of cases and is characterized by high chromosomal instability (CIN-H), microsatellite stability (MSS) and low levels of gene hypermethylation. The most frequent mutations include recurrent APC (75%), TP53 (70%) and KRAS (30%), while BRAF mutations were absent, there is increased downstream targets of WNT and MYC, elevated expression of EGFR, HER2, IGF2, IRS2, HNF4A and cyclin, and are more frequent in the distal colon^10,21^.

The CMS3 subtype or metabolic subtype (10%) is characterized by the activation of glutaminolysis and lipidogenesis and by the presence of a distinct genomic and epigenomic profile when compared to other tumors with chromosomal instability. This occurs due to the presence of a mixed CIMP-H (20%), MSI-H (15%), hypermutation (30%) and CIN-H (54%), at the mutational level frequent mutations are found in KRAS and APC and less frequently in TP53 and BRAF. The most common morphology is papillary and they are located at the proximal and distal level of the colon^14^.

Comprising 25% of cases, the CMS4 subtype, known as mesenchymal, is characterized by the presence of tumors that exhibit activation of pathways related to epithelial-mesenchymal transition (EMT) and stemness (TGF-β and integrin signaling) and overexpression of genes involved extracellular matrix remodeling, stromal invasion and angiogenesis, complement-associated inflammation. The marked infiltration of stromal cells in the tumor microenvironment is typical in these tumors, as well as CIMP-H and MSI-H are frequently associated with high chromosomal instability, although rarely hypermutated. Mutations in APC, TP53 and KRAS are common, as well as rare mutations in BRAF. From the histological point of view, they have a desmoplastic characteristic with high stroma and have a worse prognosis when compared to other subtypes^10^.

There is also a subgroup that is not possible to be included in any of those described above, which make up 10-15% of the total number of tumors and have mixed characteristics, reflecting tumor heterogeneity^10^.

In recent years, some studies have shown that CRC presents clinically relevant molecular heterogeneity related to several genetic and non-genetic mechanisms. The identification of molecular subtypes helped to demonstrate new treatment strategies for selected groups of patients, the so-called “target therapies”. As the presence of mutations in the KRAS or NRAS genes that allowed the identification of the refractoriness of this subgroup of patients with the use of therapies with EGFR inhibitors; as well as the presence of “wild-type” tumors, with no mutation in the KRAS, NRAS, BRAF and PIK3CA/PTEN genes would be responsive to EGFR inhibitors. Another example would be patients with the BRAF V600E mutation, who have a worse prognosis, but who respond to combined treatment with anti EGFR in association with a BRAF inhibitor. As well as other molecular targets such as HER-2 amplification, where patients may be sensitive to anti HER-2 blockade; as well as hypermutated RCC patients such as MSI-H and POLE who are particularly responsive to the use of immune checkpoint inhibitor treatments. And finally, patients with mesenchymal phenotype who exhibit immunosuppressive mechanisms that can be removed through treatments combined with immunotherapy^8^.

The initial treatment strategy for advanced disease always relies on the molecular profile of the disease, the location of the tumor and the patient’s performance status as key features.

### Genetics of colorectal cancer of the primary lesion and its correspondence in metastases

Around 20% of patients are diagnosed with advanced disease, its main sites of metastases are liver, lung, peritoneum, bone and central nervous system with greater rarity. Numerous comparative studies of tumor sequencing of primary lesions and metastases have been performed and a high degree of agreement has been observed^12^. These data reinforce the view that a better understanding of molecular alterations and their heterogeneity can improve the outcome of the treatment of these patients. In one study^19^, the analysis of KRAS, NRAS, BRAF, PIK3CA and TP53 genes in 84 patients with colorectal cancer was reported. As a result, it was observed that the frequency of mutations in the KRAS, NRAS and PIK3CA genes were similar in metastatic tumors versus primary tumors; TP53 mutations were more frequent in metastatic versus primary tumors (53 versus 30%, respectively), while BRAF mutations were significantly less frequent (1.9 versus 7.7%). In this same study, discordant mutations in KRAS/NRAS and BRAF were not observed; the only private mutations, defined as mutations seen only in the primary or metastatic tumor, were seen at the level of the APC, PIK3CA, SMAD4 and TP53 genes. These findings have supported the view that genetic alterations that occur early during the genesis of colorectal cancer, such as APC, KRAS, NRAS and BRAF mutations, are maintained during the tumor evolution process until the final level of tumor metastases^2,7^.

It was through a meta-analysis of 61 clinical studies with approximately 3,565 patients with metastatic RCCs that it was demonstrated:

1. A very high median of agreement of biomarkers for KRAS (93%), NRAS (100%), BRAF (99.4% ), PIK3CA (93%);
2. A pooled discordance of 8% for KRAS, 8% for BRAF and 7% for PIK3CA.

These findings further support the maintenance of key driver mutations in patients with colorectal cancer who undergo metastatic spread^1^.

### Liver metastases

The liver is one of the most frequent sites of dissemination of colorectal cancer. Liver metastases may be amenable to local surgical treatment or through procedures such as therapeutic ablation, which leads to a gain in survival for this subpopulation. A possible link between genomic characteristics and outcomes in patients with metastatic colorectal cancer undergoing resection of liver metastases was evidenced. Some studies have shown that the presence of double mutation RAS/TP53 in tumors located in the right colon (31% of patients) had a lower 5-year overall survival of 12%, when compared to 55% in the subgroup of patients with TP53 wild type^6^.

In 5.1% of patients with metastatic colorectal cancer, the BRAFV600E mutation is observed and it has been associated with a worse prognosis in patients undergoing surgical treatment for CRC liver metastasis. The same was not observed in non-V600E mutations^14^.

In a study of 935 patients, Datta et al. evaluated patients with metastatic RCC and showed that co-alteration of oncogenic TP53 in association with KRAS, NRAS or BRAF mutations were related to worse survival compared to alterations in genes alone^7^.

Very similar results were found in another study by Kawaguchi et al. in which the possible relationship between the somatic gene mutation profile and the outcome was analyzed in 507 patients with metastatic RCC who underwent resection of liver metastases. Double or triple mutations in RAS, TP53 and SMAD4 are associated with worse overall survival and recurrence-free survival after surgical treatment when compared to mutations in only 1 or none of these genes. It is important to point out that this was a retrospective study, generating hypotheses of possible prognostic factors^13^.

## CONCLUSION

With the development of treatments in metastatic disease and the use of targeted therapies and their biomarkers, it was possible to evaluate them within clinical studies both in the primary tumor and in the correspondence of metastases. And these mutational status concordances data for KRAS, NRAS, BRAF and PIK3CA have been evaluated in multiple clinical studies in over 3500 patients as described above.

As an example of the importance of analyzing the molecular profile of metastatic disease is the use of EGFR inhibitors, which are effective in a subset of wild-type allRAS RCC. However, it is known that after an initial response, resistance mechanisms may occur, evolving to disease progression. It was through molecular analyzes in clinical studies that the acquisition of secondary KRAS mutations was most frequently identified; mainly through analysis of circulating tumor DNA. In fact, in the future, the use of circulating tumor DNA will be an important tool for defining subsequent treatments, given the possibility of assessing the resistance profile, with a minimally invasive test. We are currently awaiting robust studies and clinical and economic applicability.
